# Salivary Biomarkers of Oxidative Stress and Inflammation in Stroke Patients: From Basic Research to Clinical Practice

**DOI:** 10.1155/2021/5545330

**Published:** 2021-04-07

**Authors:** Mateusz Maciejczyk, Marzena Bielas, Anna Zalewska, Karolina Gerreth

**Affiliations:** ^1^Department of Hygiene, Epidemiology and Ergonomics, Medical University of Bialystok, 2C Adama Mickiewicza Street, 15-022 Bialystok, Poland; ^2^Department of Family Medicine, Poznan University of Medical Sciences, 49 Przybyszewskiego Street, 60-355 Poznan, Poland; ^3^Experimental Dentistry Laboratory, Medical University of Bialystok, 24A Marii Sklodowskiej-Curie Street, 15-276 Bialystok, Poland; ^4^Department of Risk Group Dentistry, Chair of Pediatric Dentistry, Poznan University of Medical Sciences, 70 Bukowska Street, 60-812 Poznan, Poland

## Abstract

Cerebral stroke is a serious worldwide health problem, as can be seen by the global epidemic of the disease. In this disorder, when the blood flow is compromised by ruptures or blocked arteries, sudden death of neurons is observed as a result of a lack of oxygen and nutrients. Numerous severe problems and frequent complications also exist in stroke patients; therefore, there is an urgent need to develop new therapeutic, diagnostic, and prognostic methods for the disease. At present, the diagnosis of stroke is based on a neurological examination, medical history, and neuroimaging, due to the fact that rapid and noninvasive diagnostic tests are unavailable. Nevertheless, oxidative stress and inflammation are considered key factors in stroke pathogenesis. Oxygen free radicals are responsible for oxidation of lipids, proteins, and DNA/RNA, which in turn contributes to oxidative damage of the brain. Toxic products of the oxidation reactions act cytostatically on the cell by damaging cell membranes and leading to neuronal death by apoptosis or necrosis. Thus, it seems that redox/inflammatory biomarkers might be used in the diagnosis of the disease. Nowadays, saliva is of increasing interest in clinical laboratory medicine. Redox biomarkers could be obtained easily, noninvasively, cheaply, and stress-free from saliva. This minireview is aimed at presenting the current knowledge concerning the use of salivary biomarkers of oxidative stress and inflammation in the diagnosis and prognosis of stroke.

## 1. Introduction

At present, cerebral stroke is a serious worldwide health problem, as can be seen by the global epidemic of the disease. It was established that between 1990 and 2010, the age-standardized incidence of cerebral stroke increased by 12% in middle- and low-income countries [1, 2]. Furthermore, the incidence and the prevalence of disease rise with life expectancy [3]. A cerebrovascular accident is the third main cause of serious long-term disability, the second leading cause of death, and an essential reason of depression and dementia [4].

In cerebral stroke, when the blood flow is compromised by a ruptured or blocked artery, sudden death of neurons is observed as a result of a lack of oxygen and nutrients [4, 5]. Therefore, the stroke classification includes ischemic stroke (caused by an obstruction of a blood vessel by a thrombus) and hemorrhagic stroke (resulted from a ruptured blood vessel) [6]. Hemorrhagic strokes include subarachnoid hemorrhage (SAH; bleeding between the inner and outer layers of tissue covering the brain within the subarachnoid space) as well as intracerebral hemorrhage (ICH; bleeding within the brain) [6, 7]. Ischemic strokes are considered to be much more frequent, since they represent 87% of all cases [5]. Risk factors for stroke include age, sex, family history of stroke, obesity, hypertension, diabetes, dyslipidemia, inflammatory states, atrial fibrillation, hypercoagulability, hyperhomocysteinemia, hormone replacement therapy, smoking, alcohol use, lack of physical activity, asymptomatic carotid stenosis, and cardiac disease [2, 6–8]. Although strokes have primarily vascular pathology, they affect the blood vessels in the brain as well as the entire body. It is very disturbing seeing that the incidence of stroke complications is very high and can affect between 40% and 96% of rehabilitated or hospitalized individuals [3]. Stroke-related disorders contain epileptic seizures, limb paresis, deep vein thrombosis, headaches, and urinary tract infections [3, 6, 9].

Currently, rapid diagnostic tools for stroke patients are not available [5, 10]. The diagnosis of stroke is based on a neurological examination, medical history, and brain neuroimaging [5, 11]. Development of quick diagnostic methods would be helpful in the optimization of patient management to improve stroke outcomes. In particular, laboratory biomarkers of stroke are still being sought. Special attention has been paid to redox and inflammatory biomarkers, because oxidative stress and increased cytokine formation play a key role in stroke pathogenesis [12, 13]. Indeed, oxygen free radicals are responsible for the oxidation of neuronal lipids, proteins, and nucleic acids, which in turn contributes to oxidative damage of the brain [3, 14–16].

Saliva is gaining increasing interest in laboratory medicine [17–21]. It appears to be an excellent diagnostic material and can be successfully used as an alternative to blood and urine. The ease of sample collection and transport, noninfectivity, and high stability of the analyzed compounds make this biological fluid highly useful [17, 19, 21]. Since there are severe problems and frequent complications in stroke, there is an urgent need to develop new therapeutic, diagnostic, and prognostic methods. Salivary redox biomarkers may be the new hope in stroke diagnostics [22–26]. Therefore, this review paper is aimed at presenting the current knowledge concerning the use of salivary markers of oxidative stress and inflammation in the diagnosis and prognosis of stroke.

## 2. Oxidative Stress, Inflammation, and Stroke

Oxidative stress is a condition in which excessive reactive oxygen (ROS)/nitrogen (RNS) species activity or impaired redox signaling leads to oxidation of cellular biomolecules [17, 27, 28]. Although free radicals are essential for many physiological processes (such as cellular growth/differentiation, mitogenic response, extracellular matrix remodeling, and apoptosis), excessive production of ROS impairs gene expression, cytokine/chemokine production, and cellular metabolism [27–29]. ROS overproduction increased oxidation to miscellaneous biomolecules, hence changing several signaling pathways and promoting cellular injury and neuronal death [27, 29, 30]. The brain is especially vulnerable to oxidation by ROS. It is one of the most metabolically active organs with an elevated oxygen demand. The activity of antioxidant enzymes in the brain is also relatively low, and the regenerative capacity of neurons is very limited [17, 31].

**Figure 1:**
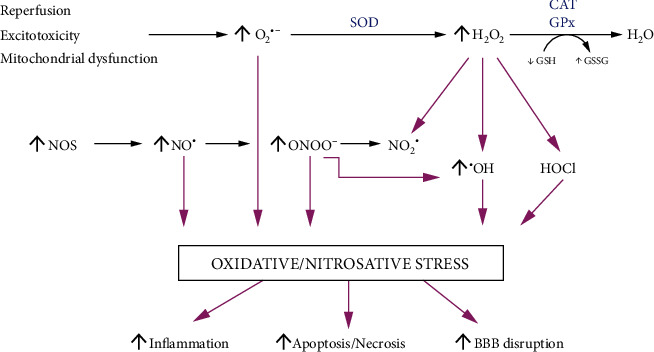
Redox imbalance in stroke brain. In stroke patients, there is an increased production of superoxide radical and peroxynitrite, which leads to intensified oxidative and nitrosative brain damage. This condition results not only in the increased synthesis/secretion of proinflammatory cytokines but also in blood-brain barrier (BBB) disruption and enhanced neuronal apoptosis. Abbreviations: CAT: catalase; GPx: glutathione peroxidase; GSH: reduced glutathione; GSSG: oxidized glutathione; SOD: superoxide dismutase.

Redox imbalance has a key role in stroke pathogenesis (Figure 1) [3, 14, 15, 32]. Oxidative stress impairs lipid raft turnover, leads to mitochondrial dysfunction, and disturbs cross-communication in the neurovascular coupling [14, 33]. The most important oxidants that cause brain injury are superoxide radical anion (O_2_^−^), hydrogen peroxide (H_2_O_2_), and nitric oxide (NO) [14, 33, 34]. The effects of ROS action include excessive lipid peroxidation, altered function of receptors, ion channels, and other membrane proteins, which impair cell membrane fluidity and permeability [33–35]. Ischemic brain injury also disturbs the blood-brain barrier (BBB) leading to leukocyte infiltration and edema [36, 37]. Under these conditions, inflammatory reactions are intensified [38, 39]. Additionally, glucose and oxygen deprivation inhibits ATP synthesis and blocks the activity of the sodium-potassium pump (Na^+^/K^+^-ATPase); this subsequently causes the inflow of Ca^2+^ ions into the cell [33–35]. Increased concentrations of Ca^2+^ activate cyclooxygenases (COX-1 and COX-2) and phospholipase A2, which not only increases ROS production but also enhances glutaminergic neurotransmission [22, 36]. In ischemic stroke, increased levels of sodium, calcium, and adenosine diphosphate (ADP) also result in the overproduction of mitochondrial ROS (mROS) [5, 24]; this leads to neuronal cell death by apoptosis [40, 41]. However, oxidative stress is also observed in hemorrhagic stroke. In this type of stroke, the major sources of ROS are mitochondrial dysfunction, activation of inflammatory cells, degradation of hypoxanthine, and conversion of arachidonic acid to prostanoids [14, 42]. Cerebral oxidative stress also exaggerates the inflammatory response through the NF-*κ*B (nuclear factor kappa-light-chain-enhancer of activated B cells) signaling; thus, the expression of several cytokines (IL-1, IL-2, IL-6, and TNF-*α*), chemokines (CCL2, CCL3, CCL5, and CXCL2), and growth factors (G-CSF, VEGF, and IGF-1) increases [5, 39, 43–45]. Conversely, nitric oxide also has a principal role in stroke complications; its cytotoxicity damages cellular DNA, blocks mitochondrial activity, and enhances nitrosative damage by peroxynitrite (ONOO^−^) formation [46, 47]. Generally, NO is produced by endothelial nitric oxide synthase (eNOS); however, in inflammatory conditions, inducible nitric oxide synthase (iNOS) is overexpressed by smooth muscle cells and macrophages, generating high amounts of NO [48]. Nitric oxide is also produced by neuronal NO synthase (nNOS) [36, 49]. When O_2_^−^ collides with NO, both molecules quickly respond to form the highly reactive ONOO^−^ [50]. A superoxide anion can also be dismutated to the more stable H_2_O_2_ in the reaction catalyzed by superoxide dismutase (SOD). O_2_^−^ is one of the most vital ROS in the central nervous system, since it has the potential to injure the ROS-producing and neighboring cells [48, 51]. Superoxide is a product of xanthine oxidase (XO) and nicotinamide adenine dinucleotide phosphate (NADPH) oxidase (NOX) activity as well as a by-product in the mitochondrial respiratory chain reactions [38, 48, 52, 53]. Interestingly, when the level of O_2_^−^ is also increased, the NO radical has dual actions. NO interferes with SOD by decreasing its antioxidant effect [48].

## 3. Saliva as a Source of Redox and Inflammatory Biomarkers

A biomarker is an indicator of the normal biologic and pathologic processes, as well as the pharmacological response to a drug, which can be objectively measured [54]. The ideal biochemical marker of stroke should have high specificity (i.e., be able to distinguish stroke from other neurological diseases, such as combined migraine or multiple sclerosis) and be identifiable in readily available biological material, and its concentration should correlate with that in cerebrospinal fluid. In addition, the biomarker should be identifiable within a short time after stroke onset and be a prognostic indicator for the efficacy of thrombolytic therapy. However, no biomarker known so far has a high enough sensitivity and specificity to be useful in stroke diagnostics [5, 10, 11].

Oxidative stress plays a key role in stroke pathogenesis [3, 14, 15]; as a result, redox biomarkers have been postulated for diagnosis and monitoring of disease progression [22–25]. Saliva, which faithfully reflects the composition of blood or cerebrospinal fluid, has gained increasing interest in redox diagnostics. This biofluid is collected in a simple, noninvasive, and painless way, without the involvement of medical staff. Saliva is also a noninfectious diagnostic material, and its components tend to be more stable than those present in the blood serum [17, 19, 21, 55–57].

**Figure 2:**
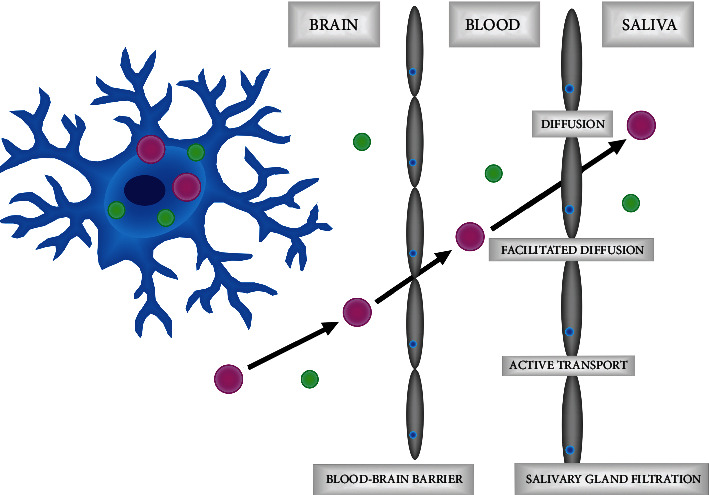
Transport of antioxidants and cellular oxidation products from the brain to the saliva. Antioxidants (green molecules) as well as protein, lipid, and DNA oxidation products (pink molecules) are transported to the salivary glands via an intracellular or extracellular route. The intracellular route includes passive transport (diffusion, filtration, and facilitated diffusion) and energy-demanding transport (active transport), while the extracellular route occurs by ultrafiltration or through damaged cellular membranes.

Saliva is the secretion of large (submandibular, parotid, and sublingual) and small salivary glands. The submandibular glands are mainly responsible for resting salivary secretion, whereas the parotid glands produce the most saliva during stimulation [58, 59]. 99% of the salivary composition is water, while the rest is inorganic (sodium, potassium, chlorides, carbonates, and phosphates) and organic (proteins, mucins, and lipids) [59–61]. Antioxidants and oxidation products are also very important constituents of saliva [62]. Antioxidant enzymes (salivary peroxidase system (Px), lactoperoxidase, myeloperoxidase (MPO), glutathione peroxidase (GPx), or catalase (CAT)) as well as low molecular weight nonenzymatic antioxidants (uric acid (UA), reduced glutathione (GSH), albumin, or vitamin C) are the first line of defense against oxygen free radicals [62–64]. The oral cavity is the only place in the body that is exposed to so many environmental factors such as air pollutants, xenobiotics, stimulants, or dental materials/treatments [65–67]. Although many external factors can affect the redox homeostasis of saliva, its composition is not significantly different from blood serum and plasma [68–70]. Antioxidants, oxidation products, and cytokines, chemokines, and growth factors found in the blood are transported to the salivary glands via an intracellular or extracellular route (Figure 2). The intracellular route includes passive transport (diffusion, filtration, and facilitated diffusion), energy-demanding transport (active transport), or pinocytosis. In contrast, the extracellular route can occur by ultrafiltration or through damaged cellular membranes [61, 71]. Thus, blood concentrations of many redox biomarkers correlate with their salivary content [63, 68–70, 72]. In addition, some compounds are also produced in salivary glands, such as salivary peroxidase and salivary lactoperoxidase [64]. Although still little is known about the biokinetics of OS biomarkers found in saliva, their passage into the oral cavity depends on their molecular weight, solubility in water and lipids, degree of ionization, and half-life of the compound [73, 74]. Therefore, direct analysis of the rate of salivary ROS/RNS formation is practically impossible. To assess redox status using saliva, protein, lipid, and DNA/RNA oxidation products are mainly utilized [75–78]. These compounds are formed by the reaction of free radicals with cellular biomolecules and are much more durable than ROS and RNS [79]. Furthermore, they provide more information about the consequences of oxidative damage to the human body [79, 80]. Antioxidant enzyme activity and concentration of nonenzymatic antioxidants are also assessed in saliva samples [63, 81, 82]. Both nonstimulated (NWS) and stimulated (SWS) whole saliva is used in diagnostics.

Salivary redox/inflammatory biomarkers are used to evaluate not only oral [83–87] but also several systemic diseases. Indeed, the diagnostic utility of salivary oxidative stress/inflammatory indexes has been demonstrated in patients with obesity [88–91], insulin resistance [88, 92], diabetes [93, 94], hypertension [95, 96], metabolic syndrome [88, 97], chronic kidney disease [98–100], heart failure [101–103], psoriasis [82, 104], Hashimoto's disease [105, 106], Alzheimer's disease [17, 77, 107], or cancer [108–110]. Recent findings also indicate the clinical usefulness of salivary/inflammatory redox biomarkers in stroke diagnostics [22–26, 111].

## 4. Materials and Methods

The articles were selected from searches of PubMed, Web of Science, and Google Scholar databases. All papers were published in English between 1950 and 2021. We included the clinical studies with human subjects. In order to find all of the relevant articles, the databases were searched using the following keywords: “stroke”, “oxidative stress” or “OS”, “antioxidant”, “inflammation”, “biomarker”, and “saliva” in various combinations. Specific inclusion and exclusion criteria are shown in Table 1. Finally, only six original papers were taken into consideration (Table 2).

## 5. Salivary Redox and Inflammatory Biomarkers in Stroke Patients

### 5.1. Salivary Antioxidants

As a result of evolution, aerobic organisms have evolved mechanisms to prevent/eliminate the harmful effects of ROS and their metabolites [112]. Antioxidant mechanisms inhibit the formation of free radicals, reduce the interactions of ROS with cellular components, and interrupt free radical chain reactions. Under physiological conditions, there is a balance between the production of ROS and their neutralization. Nevertheless, in stroke patients, the oxidoreductive status shifts in favor of oxidative reactions [33].

In studies conducted to date, both enzymatic and nonenzymatic antioxidants have been assessed in the saliva samples of stroke patients. In general, an enhanced antioxidant barrier was demonstrated in both nonstimulated (↑Px, ↑CAT, ↑SOD, and ↑UA) and stimulated (↑Px, ↑CAT, and ↑UA) saliva, suggesting an adaptive response of the body to the increased formation of ROS in stroke patients [22, 23]. However, salivary glutathione content (↓GSH) was significantly reduced in stroke cases as compared to the healthy controls. Importantly, changes in saliva of stroke patients generally reflect variations in the blood. There were no significant differences in salivary antioxidant defense between ischemic and hemorrhagic strokes [22].

The decrease in GSH levels in stroke patients is considered to be of critical clinical importance. Glutathione (gamma-glutamyl-cysteinyl-glycine) is the most prevalent low molecular weight thiol compound found in the central nervous system [113, 114]. GSH not only scavenges oxygen free radicals but also maintains the thiol groups of proteins in a reduced state, participates in cell proliferation and apoptosis, and acts as a specific modulator in glutamatergic neurotransmission [113, 115]. GSH deficiency in cerebral stroke leads to oxidation of membrane proteins and lipids, increased permeability of mitochondrial membranes to Ca^2+^ ions, and impaired oxidative phosphorylation [116, 117]. GSH is also found in saliva and significantly correlates with its plasma content [107, 118, 119]. Given that changes in plasma glutathione may reflect the thiol status of the brain, assessment of the salivary GSH level may have an important diagnostic value [120, 121]. In clinical studies, it has been shown that reduced glutathione content was significantly lower in both serum and saliva of stroke patients. GSH SWS with sensitivity and specificity equal to 100% differentiates the study group from healthy controls (AUC 1.0) [22]. These results were confirmed by the study of Al-Rawi et al. [23]. They showed that both salivary (accuracy 81%, AUC 0.669) and serum reduced glutathione (accuracy 80%, AUC 0.912) differentiate between healthy and stroke individuals [23]. Using multifactorial regression analysis, it was proven that salivary glutathione content does not depend on gender, age, and salivary gland function. Additionally, it has been shown that salivary GSH correlates positively with dynamic balance abilities in the BBS scale and cognitive function in the ACE III (Addenbrooke's Cognitive Examination III) scale [22]. Therefore, salivary glutathione may be a potential biomarker not only for the diagnosis of stroke but also for the monitoring of disease progression. As we have shown in our previous studies, salivary GSH is a noninvasive indicator of a cognitive function in elderly people. It can also differentiate between different types of dementia [107, 118].

The compound responsible for up to 70-80% of the antioxidant properties of saliva is uric acid [63, 64]. UA is the end product of purine metabolism, which reacts with superoxide anion, hydrogen peroxide, hydroxyl radical, and peroxynitrite [122, 123]. UA is considered the main antioxidant of saliva and blood plasma [63, 64]. Nevertheless, at high concentrations, UA has a strong prooxidant activity. Indeed, UA can generate free radicals by a reaction with peroxynitrite, enhancing ROS-generating enzymes (e.g., NADPH oxidase and NOX) or alkylating cellular biomolecules [123, 124]. The compound passes from plasma by passive diffusion, and its salivary content generally reflects the blood UA concentration. A high salivary-blood correlation coefficient for UA has been demonstrated not only in healthy individuals [125] but also in patients with chronic kidney disease [57, 75, 126] or obesity [89, 97, 127]. In stroke patients, UA levels were found to be significantly higher in both nonstimulated and stimulated saliva as well as blood serum when compared to the healthy controls [22, 23]. Importantly, salivary UA content does not depend on gender or salivary flow rate which increases the diagnostic utility of the biomarker [22]. Salivary UA levels were also significantly higher in patients with hypertension, diabetes mellitus, and ischemic heart disease [23, 72, 95]. These conditions significantly increase the risk of stroke development and exacerbate patient mortality [2, 128]. Al-Rawi et al. [23] showed that an increase in salivary UA content in ischemic stroke and stroke-related diseases may reflect a separate disease process (such as atherosclerosis) as well as enhance xanthine oxidase (XO) activity catalyzing the UA formation from hypoxanthine. In another study, salivary UA with high sensitivity (70%) and specificity (63.33) differentiated ischemic and hemorrhagic stroke from healthy controls (AUC 0.7611) [22]. These observations are confirmed by Al-Rawi et al. [23] in ischemic stroke cases (accuracy 89.3%, AUC 0.95). Interestingly, the diagnostic utility of salivary UA was significantly higher than the serum UA assessment (accuracy 89.3%, AUC 0.927) [23]. Moreover, the authors showed that salivary UA was accurate in predicting stroke in patients with hypertension (AUC 0.66) and type 2 diabetes (AUC 0.709) [23]. It should be recalled that in a population with a very high cardiovascular risk, elevated UA concentration is an independent predictor of patient mortality [128, 129]. Associations of salivary UA with other redox biomarkers are also worth noting. NWS UA correlated positively with SOD and MDA, indicating a direct effect on the systemic oxidative stress level [23]. Therefore, salivary UA can be used to assess central redox homeostasis in stroke patients.

High clinical utility in stroke diagnostics has also been demonstrated for salivary SOD [22, 23]. Indeed, both NWS (accuracy 89.3%, AUC 0.918) and serum SOD (accuracy 80%, AUC 0.838) significantly differentiate healthy individuals from those with ischemic stroke [23]. The increase in SOD activity should not be surprising because the generation of superoxide radical anions usually occurs during reperfusion [14]. O_2_^−^ is produced in the one-electron reduction of molecular oxygen (O_2_) formed by electron leakage in the mitochondrial respiratory chain and also during phagocyte activation and oxidation of xanthine to hypoxanthine in the UA production cycle. O_2_^−^ has been shown to play a key role in oxidative damage during ischemia as well as after reperfusion [48, 51]. Unfortunately, due to its half-life, assessment of the superoxide radical anion is difficult and must be based on the evaluation of SOD activity [130]. A potential link between increased O_2_^−^ production and uric acid synthesis may be evidenced by the positive correlation between salivary SOD and UA [23]. Salivary SOD is also a potential predictor of stroke in hypertensive (AUC 0.679) and ischemic heart disease (AUC 0.661) cases, which confirms the diagnostic utility of this biomarker [23].

Increased salivary peroxidase activity was also observed in both NWS and SWS of stroke patients [22]. Px is one of the few antioxidants produced exclusively by the salivary glands [63, 64, 131]. The salivary peroxidase system not only is responsible for the decomposition of hydrogen peroxide but also catalyzes the oxidation of thiocyanates (SCN^−^), bromides (Br^−^), and chlorides (Cl^−^), inhibiting the glucose uptake, as well as growth of the cariogenic bacteria in the oral cavity [64, 131]. Thus, the increase in salivary Px activity suggests a strengthening of the antioxidant barrier only in the salivary glands of stroke patients [22].

### 5.2. Salivary Redox Status

In stroke patients, the salivary antioxidant barrier is generally strengthened as evidenced by an increase in antioxidant enzyme activity (↑Px, ↑CAT, and ↑SOD) and salivary UA levels [22, 23]. Nevertheless, a decrease in salivary/serum glutathione concentrations was also observed in this group of patients. GSH is the most important intracellular antioxidant which significantly affects the oxidative-reductive status of the body [132, 133]. Therefore, Gerreth et al. [22] assessed salivary total antioxidant (TAC) and oxidant (TOS) capacity, which evaluates the resultant ability of a biological system to counteract oxidative stress and cellular oxidative damage. TAC represents the total free radical scavenging activity and provides much more information than assessing the concentration of individual antioxidants [134–136]. Indeed, the interaction between different ROS scavengers gives a greater antioxidant potential than any of the compounds alone. Importantly, salivary and urinary TAC generally reflect the systemic antioxidant barrier, thereby indicating the body's protection against oxidative stress [98, 137].

Salivary TAC was not significantly different in both nonstimulated and stimulated saliva of stroke patients when compared to controls [22]. This suggests a balancing of salivary enzymatic (↑Px, ↑CAT, and ↑SOD) and nonenzymatic (↓GSH) mechanisms despite a significant increase in salivary uric acid levels. However, does this condition prevent oxidative stress in stroke patients? Salivary TOS and oxidative stress index (TOS/TAC ratio) were significantly higher in stroke cases indicating a shift in redox balance in favor of oxidative reactions [22]. Indeed, TOS expresses the total amount of oxidants in the analyzed sample, while OSI is considered the gold predictor of oxidative stress [82, 138]. SWS TOS seems also to have a potential diagnostic utility. Using ROC (receiver operating characteristic curve) analysis, it was shown that this biomarker differentiates (with high sensitivity (86.67%) and specificity (83.33%)) stroke patients from healthy controls (AUC 0.8822). Importantly, salivary TOS levels are not dependent on sex, age, or salivary gland secretory activity [22].

### 5.3. Salivary Oxidation Products

The effects of oxidative stress on the body are mainly assessed by evaluating the biomarkers of cellular oxidative damage [27, 139, 140]. Oxidative injury to macromolecules is caused by ROS with high oxidizing capacity and includes lipid peroxidation, oxidation of amino acid residues/prosthetic groups of enzymes, cross-linking of proteins, transformation of nitrogenous bases, and double-strand breaks in the DNA [15, 141]. Protein, lipid, and DNA oxidation products can exhibit tissue and organellar specificity, provide information about the chemical and biological nature of the oxidant, and help in the diagnosis of various systemic diseases [79, 142, 143]. The great interest in the possible diagnostic and prognostic applications of the oxidized-modified biomolecules is based on the proven involvement of redox imbalance in the biochemical and clinical complications of stroke [14, 144, 145].

Excessive oxidation of salivary proteins (↑AOPP, ↑PC, and ↓total thiols) and lipids (↑MDA, ↑LOOH) was demonstrated in patients with ischemic and hemorrhagic stroke [22–24]. Of particular importance are biomarkers assessed in stimulated saliva, which, with very high sensitivity (AOPP: 96.67%, LOOH: 93.33%) and specificity (AOPP: 93.33%, LOOH: 90%), differentiate stroke patients from healthy controls (AOPP AUC 0.9911, LOOH AUC 0.9833) [22]. However, the diagnostic usefulness of these biomarkers was significantly lower in nonstimulated saliva (AOPP AUC 0.8711, LOOH AUC 0.8567) [22]. The best known free radical process is lipid peroxidation, in which the oxidation of unsaturated fatty acids occurs with the formation of peroxides of these compounds. Especially dangerous is the production of aldehydes such as malondialdehyde (MDA), which is the best-known biomarker of lipid peroxidation [146, 147]. MDA levels were assessed not only in the stroke subjects but also in stroke-related diseases such as hypertension, type 2 diabetes, and ischemic heart disease [23, 148–150]. Al-Rawi et al. [23] showed that MDA concentrations were significantly higher in the saliva of stroke patients followed by the serum levels. This indicates neuronal redox imbalance due to the free radical-induced damage [17, 118]. Lipid peroxidation occurs particularly easily because the brain is a rich source of polyunsaturated fatty acids [17, 31]. Interestingly, increased levels of salivary MDA were also noted in patients from stroke risk groups [23]. Salivary MDA with a higher accuracy rate (92%) than serum MDA (81%) differentiates healthy individuals from those with ischemic stroke (salivary AUC 0.969, serum AUC 0.885). This biomarker also showed high diagnostic utility in differentiating stroke subjects from hypertensive (AUC 0.646) and ischemic heart disease (AUC 0.705) patients [23]. MDA reacts with structural proteins, resulting in altered cell membrane fluidity, modification of the membrane-associated enzyme activity, alterations of membrane channels and receptors, and uncoupling of the transmembrane transport in stroke cases [148, 151]. Moreover, salivary MDA correlated positively with UA content in saliva. Al-Rawi et al. [23] showed that salivary MDA is directly affected by systemic oxidative stress, because MDA levels were elevated in ischemic stroke patients and patients with stroke-related diseases. Unfortunately, there are some limitations to the use of salivary protein and lipid oxidation products as biomarkers [73, 99, 152]. Primarily, the salivary content of these compounds depends on the secretory function of the salivary glands. In some patients with stroke, there is a decreased salivary secretion (hyposalivation), which is responsible for changes in the quantitative and qualitative composition of saliva. This is confirmed by the strong negative correlation not only with the salivary flow rate but also with total protein content and salivary amylase activity [22, 24]. Secondly, the oxidation of salivary/plasma proteins and lipids intensifies significantly with age [22, 118, 125]. Thus, it is necessary to develop laboratory reference values for salivary redox biomarkers in different age ranges of the population.

### 5.4. Salivary Glycation Products

Proteins undergo many posttranslational modifications. These include nonenzymatic glycosylation (glycation), which involves a reaction between a reducing monosaccharide (usually glucose) and the primary free amino group of the terminal amino acid in the protein [153, 154]. Although it is a process that occurs spontaneously in all living organisms, tissue damage caused by the accumulation of advanced glycation end products (AGEs) is one of the causes of many systemic diseases, including diabetes, hypertension, cardiovascular disease, kidney failure, neurodegenerative disorders, and also stroke [13, 155–159]. Therefore, increased rates of protein glycation are thought to be a predisposing factor for stroke and its related complications [7, 13]. Glycation and oxidation mutually intensify each other, so that these processes are collectively referred to as glycoxidation [153, 160].

Previous studies have demonstrated increased salivary levels of both early (↑Amadori products) and late (↑AGE) glycation products in both ischemic and hemorrhagic stroke patients [22, 24]. The content of protein glycoxidation products was also significantly higher in both nonstimulated (↑dityrosine, ↑kynurenine, ↑N-formylkynurenine, and ↓tryptophan) and stimulated (↑N-formylkynurenine, ↓tryptophan) saliva of stroke patients as compared to the healthy controls. Only the tryptophan concentration was significantly reduced [24]. Nevertheless, it is well known that protein glycoxidation affects the quenching of tryptophan (Trp214) fluorescence due to the partial opening of hydrophobic pockets in the glycation-modified proteins [139, 161]. Thus, in stroke patients, salivary protein glycation and glycoxidation are significantly intensified. Interestingly, salivary AGE with high sensitivity (NWS: 80%, SWS: 73.33%) and specificity (NWS: 76.67%, SWS: 66.67%) differentiated the study group from control subjects (AUC NWS: 0.8078, AUC SWS: 0.7522) [22]. However, in stroke patients, the rate of protein glycoxidation greatly depends on the salivary gland secretory function [22, 24]. This precludes the use of these biomarkers in clinical laboratory medicine. The key involvement of carbonyl and oxidative stress has been confirmed in the salivary gland dysfunction of stroke patients [24, 102]. The content of protein glycation (NWS: ↑Amadori products, ↑AGE; SWS: ↑AGE) and glycoxidation (NWS: ↑dityrosine) products was significantly higher in the saliva of stroke patients with hyposalivation when compared to stroke cases with normal salivary secretion and healthy controls. The intensity of protein oxidation was also significantly higher in this group of patients (NWS: ↑PC vs. control group; SWS: ↓total thiols vs. control group) [24]. Glycooxidatively modified proteins show loss of biological activity and have the ability to form aggregates. The resulting products inhibit the proteolytic enzymes responsible for their degradation, promoting the accumulation of altered proteins in the salivary glands [89, 102, 162]. Indeed, salivary levels of protein glycation/oxidation products correlated negatively with the secretory function of salivary glands, expressed as reduced: NWS flow rate, total protein content, and salivary amylase activity [22, 24]. These relationships were observed only in the nonstimulated saliva of stroke patients with hyposalivation and were not demonstrated in NWS and SWS of subjects with normal salivary secretion (i.e., stroke cases with normosalivation as well as healthy controls). The content of analyzed carbonyl stress biomarkers was also significantly higher in NWS of stroke patients with hyposalivation when compared to stimulated saliva, indicating an impaired secretory function of mainly the submandibular glands [24]. Under resting conditions, the submandibular glands are responsible for approximately 60-70% of total saliva production [59, 61]. Accumulation of protein oxidation/glycation products in the salivary glands leads to not only stiffening of blood vessels but also hypertrophy of the extracellular matrix (ECM). Under these conditions, cross-linking of ECM components intensifies, and in particular, the number of cross-bonds in the collagen structure is increased. As a result, collagen fibers become stiffer and less susceptible to enzymatic breakdown [158, 163, 164]. Consequently, there is an impaired salivary secretion in the salivary gland ducts, as well as disrupted protein secretion into saliva [89, 110, 162, 165]. However, AGE and AOPP can also bind to receptors on the salivary gland surface, which affects several intracellular processes. Activation of the receptor for AGE (RAGE; receptor for advanced glycation end products) enhances the synthesis of proinflammatory cytokines (mainly IL-1 and TNF-*α*) stimulating the secretion of collagenases and other proteolytic enzymes [155, 158]. The activity of the extracellular matrix metalloproteinases (MMPs) is also increased, with a decrease in their tissue inhibitors (TIMPs). This disrupts ECM remodeling and exacerbates salivary gland dysfunction [166, 167]. RAGE receptor stimulation also activates the proinflammatory transcription factor NF-*κ*B, which enhances the expression of numerous cytokines and increases ROS production through upregulation of NOX activity [38, 158, 162]. Glycated proteins enhance free radical production, which potentiates AOPP/AGE formation as well as cytokine release on a positive feedback basis [38, 155, 158]. However, there are no studies evaluating the saliva-blood relationship of glycation and glycoxidation protein products in stroke patients. Therefore, evaluation of the usefulness of salivary biomarkers of protein glycation requires further clinical trials.

### 5.5. Salivary Nitrosative Stress

Nitrosative stress also plays an important role in stroke pathogenesis [7, 44, 168, 169]. Indeed, free radicals formed during reperfusion (mainly superoxide radicals) release cytokines from the mast cells and activate inducible nitric oxide synthase (iNOS). The NO formed under these conditions causes inhibition of electron flow in the respiratory chain in mitochondria as well as increases production of O_2_^−^, peroxynitrite, and prostaglandins, thereby contributing to brain injury [47, 50]. The cytotoxic effects of NO occur through nitrosylation of -SH groups of proteins, heme and nonheme iron cations, and tyrosyl residues in proteins. In addition, through S-nitrosylation and subsequent ADP-ribosylation, NO inhibits 3-phosphoglyceraldehyde dehydrogenase activity, which leads to reduction of glycolysis and a decrease in intracellular ATP concentrations [170, 171]. Nevertheless, the brain damage caused by peroxynitrite seems to be particularly dangerous. ONOO^−^ is a potent oxidant and nitrating agent that is responsible for nitrosative damage not only to the lipids and proteins but also to nucleic acids (DNA/RNA) [169–171].

Nitrosative stress biomarkers were also evaluated in nonstimulated and stimulated saliva of stroke patients [24]. There was a significant reduction in the NO level with a concomitant increase in peroxynitrite and nitrotyrosine in the NWS of stroke patients. In the stimulated saliva, only an increase in ONOO^−^ concentration was observed [24]. However, assessment of nitrosative stress markers in saliva does not appear to have a diagnostic value. Similarly to glycation and protein oxidation products, salivary nitrosative stress biomarkers indicate the degree of salivary gland hypofunction rather than the severity and progression of stroke [22, 24]. Firstly, both NO and ONOO^−^ have a very short half-life (approximately 1 s) [46, 51]. Secondly, their salivary content highly correlates with indicators of salivary gland injury, such as salivary flow rate, total protein content, and salivary amylase activity. The concentration of NO in nonstimulated saliva was also significantly lower in stroke patients with hyposalivation compared to patients with normal salivary secretion and healthy controls. A similar relationship was observed for nitrotyrosine in NWS [22, 24]. The proteins damaged in this way localize mainly to the site of formation/action of nitrating agents, which supports the previous hypotheses [172, 173]. Moreover, it should be recalled that NO has a critical role in the initiation of salivary secretion [174, 175]. Nitric oxide produced at parasympathetic nerve endings increases the concentration of calcium ions in the secretory cells of salivary glands, which activates potassium and chloride channels and thus initiates the secretion of primary saliva [174]. Importantly, a decrease in salivary NO levels was observed only in NWS of stroke patients, confirming previous reports of the impaired function of submandibular glands [24]. The reduced bioavailability of nitric oxide is probably due to the increased synthesis of peroxynitrite formed in the reaction between NO and O_2_^−^ [124]. ONOO^−^ is the main factor responsible for the formation of carbonyl groups, as well as the dimerization, nitration, and nitrosylation of amino acids and thiols [102, 165, 176]. Not surprisingly, there was a significant increase in carbonyl groups, dityrosine, and nitrotyrosine and a decrease in total thiols in the saliva of stroke patients with hyposalivation [24].

### 5.6. Other Salivary Redox Biomarkers

Recently, neuron-specific enolase (NSE) has become increasingly popular in medical laboratory diagnostics [177]. NSE (2-phospho-D-glycerate hydrolase; EC 4.2.1.11) is an acidic cytoplasmic protease highly specific for neurons and peripheral neuroendocrine cells. NSE catalyzes the dehydration reaction of 2-phospho-D-glycerate (PGA) to phosphoenolpyruvate (PEP) participating in the glycolytic energy metabolism of the brain [177]. It was shown that increased NSE activity triggers various inflammatory pathways, including the production of cytokines, chemokines, and growth factors, as well as the formation of ROS in neurons and glial cells. Therefore, NSE is also considered a marker of cellular oxidative damage [178]. Its diagnostic utility has been demonstrated in several neurodegenerative disorders, including Alzheimer's disease, Parkinson's disease, Huntington's disease, Friedreich ataxia, or amyotrophic lateral sclerosis (ALS) [178, 179]. Increased NSE levels were also observed in patients with ischemic stroke [25, 180, 181]. Interestingly, there was a highly positive correlation between NSE concentrations and infarct severity in acute ischemic brain damage [177, 182, 183]. Multiple studies have confirmed that serum NSE provides quantitative measures of oxidative brain injury as well as improves diagnosis and clinical outcome in ischemic stroke patients [177]. Therefore, it is not surprising that salivary NSE was evaluated as an alternative to blood tests. Al-Rawi et al. [25] showed that NSE is present in human whole saliva and may be used to assess neuronal damage in stroke and stroke-related conditions. Both salivary and serum NSE were significantly higher in stroke patients when compared to controls. The area under the curve (AUC) for salivary NSE was also significantly higher (0.763) compared to blood NSE (0.677). Nevertheless, NSE measured in the saliva of ischemic stroke cases showed no significant differences when compared to patients with hypertension, diabetes, and chronic heart failure [25].

### 5.7. Salivary Inflammatory Markers

In addition to ischemic/hemorrhagic damage, stroke induces a cellular-molecular immune response, targeted at the development of local and systemic inflammation [14, 39, 44]. Activated cells of the brain and vascular system are involved in the higher synthesis of cytokines, chemokines, adhesion molecules, and proinflammatory enzymes. These molecules may induce damage to the blood-brain barrier (BBB) and contribute to an enhanced inflammatory process in the ischemic area [14, 39, 44, 184]. Proinflammatory cytokines have been shown to stimulate expression of the adhesion molecules on the vascular endothelial cells, leading to an increased influx of peripheral blood cells and their infiltration into the brain [14, 39, 44, 185]. Several clinical studies have reported elevated inflammatory biomarkers: ESR (erythrocyte sedimentation rate), hematocrit, leukocytosis, CRP (c-reactive protein), and IL-1, IL-2, IL-6, or TNF-*α* [186–188]. Of particular note are the proinflammatory cytokines that have been quantified in the saliva of patients with other systemic diseases, including metabolic [189, 190] and neurodegenerative [17, 191] disorders. In patients with obesity and psoriasis, salivary cytokine levels reflect their blood content [89, 104].

In studies conducted to date, only Palm et al. [111] have evaluated salivary inflammatory factors in ischemic stroke patients. They examined the relationship of periodontal pathogens as well as local and systemic inflammation. Surprisingly, salivary matrix metalloproteinase-8 (MMP-8), MPO, and IL-1 levels, as well as the MMP-8/TIMP-1 ratio, were significantly lower in patients with ischemic stroke. However, in the study group, MMP-8 and MPO levels were statistically increased in serum samples. The study indicates that stroke patients show signs of past periodontal disease, while healthy individuals are more likely to have a current periodontal infection with endotoxemia and high levels of local inflammatory biomarkers [111]. It has also been suggested that cytokine/MMP biomarkers may represent an elevated inflammatory profile before the onset of stroke. Indeed, it is generally accepted that increased severity of inflammation is associated with higher stroke susceptibility [44, 185]. However, given the significant impact of periodontal disease on several inflammatory biomarkers [192, 193], further studies are needed to evaluate salivary cytokines, chemokines, and growth factors in stroke patients with a healthy periodontium. Of particular importance is the evaluation of salivary biomarkers by multiplex ELISA, which allows rapid screening of multiple cytokines and chemokines.

## 6. Limitations of Salivary Redox and Inflammatory Biomarkers

It must be emphasized that in spite of the undisputed advantages, salivary diagnostics also has numerous limitations. The level of salivary markers may alternate depending on sex, age, systemic hydration status, salivary flow, and local changes in the oral environment (e.g., periodontal and/or oral mucosa disorders). Additionally, different factors might influence the quantitative and qualitative composition of saliva collected for examination, e.g., age or drugs [17, 73, 194].

Individuals taking medications affecting the central nervous system, i.e., tricyclic antidepressants, neuroleptics, anxiolytics, hypnotic, and antiepileptic drugs, suffer from hyposalivation, i.e., the reduction of the nonstimulated salivary flow rate below 0.2 mL/min [195, 196]. The other pharmaceuticals comprise chemotherapeutics, analgesics, antihypertensives, and antihistamines. Some of these medicines influence electrolyte transporters, whereas others could interact with the cholinergic muscarinic receptors of the salivary glands, which results in reduced salivary secretion. Medicines, such as captopril or metformin, can also modify the antioxidant properties of saliva. Furthermore, polypragmasia and polypharmacotherapy greatly increase the disturbances of salivary gland function [17, 194–197].

Hyposalivation is very frequently detected in the elderly [125]. This might hinder saliva gathering and in consequence limit the utility of saliva as a diagnostic material. Thus, to exclude the effect of decreased salivation, the parameters assessed in this oral bioliquid should be standardized for either total protein content or salivary flow rate [17, 21]. In the older population, hyposalivation primarily affects the submandibular salivary glands, whereas stimulated saliva is mostly excreted by the parotid glands. Consequently, this shows the potential utility of stimulated saliva in noninvasive laboratory diagnostics [17, 21].

In addition, oxidative stress is inextricably connected to age. The rise in levels of oxidative damage products and changes in the antioxidant barrier have been observed substantially in various tissues, e.g., brain, blood, or saliva of older individuals [125, 198, 199]. This action is determined by the rate of ROS formation in the biological systems and by the efficacy of nonenzymatic and enzymatic antioxidants [200, 201].

Salivary redox homeostasis might also be affected by various systemic diseases [73]. In fact, it could be a peculiarly frequent issue in elderly people. Disruption in the salivary antioxidant barrier, caused by dysfunction of the salivary glands, could be noticed in patients suffering from Sjogren's syndrome [202, 203], rheumatoid arthritis [204, 205], diabetes [206, 207], or metabolic syndrome [208, 209].

Interestingly, it has been shown that dental treatment, as well as materials used, different xenobiotics, e.g., tobacco use, ethanol or drugs, physical exercises, and food (i.e., chronic high-fat/high-carbohydrate/high-protein diet) may induce oxidative stress [65–67, 165, 210]. Needless to say, the oral cavity is a unique part of the body subjected to numerous environmental factors.

Importantly, the sampling time of saliva is also very important for its diagnostics, because of diurnal variations of specific components, e.g., cortisol or protein carbonyls [77, 211]. Additionally, it needs to be stressed that the way of gathering saliva, its handling, storage, processing, and methods of analysis are highly important [17, 73, 212]. Thus, the need exists to standardize current protocols for sampling of saliva and establish reference values for salivary redox biomarkers [17, 213, 214]. The saliva should be collected at a fixed time (between 7 and 9 a.m.) after at least a 2 hr fast and an 8 hr break from medications. The patient should not perform any oral hygiene procedures (brushing teeth, rinsing mouth, or chewing gum) for at least 2 hrs prior to saliva collection. Additionally, immediately before the procedure, it is recommended to rinse the mouth with distilled water at room temperature. Saliva should be collected with the patients in a seated position, with the head slightly tilted forward. To prevent sample oxidation/proteolysis, it is advisable to expectorate the saliva into a sterile tube placed in a receptacle containing ice [73, 105, 118, 215]. The most common time required for NWS collection is 5-15 minutes, after which the samples should be centrifuged (3000 × g, 4°C, 20 min). This allows the saliva to be purified of mechanical debris and epithelial cells. Since the content of biomarkers in saliva may depend on the secretory activity of the salivary glands, it is desirable to standardize the results for total protein content [73, 105, 118, 215].

Finally, in the oral cavity, periodontal and oral mucosa diseases are the main sources of oxidative stress [65, 84, 216]. Thus, one needs to remember that the redox biomarkers should not be utilized in diagnostics of individuals with oral inflammation [217].

## 7. Summary and Perspective

Laboratory markers of stroke have been sought for many years. However, there is still no widely available, specific, and sensitive stroke biomarker, which is effective both in the early diagnosis of the disease and in monitoring of the effectiveness of treatment [5, 10, 11]. The ideal stroke biomarker should have high specificity, be easily quantifiable in biological material, and differentiate between different types of strokes.

**Figure 3:**
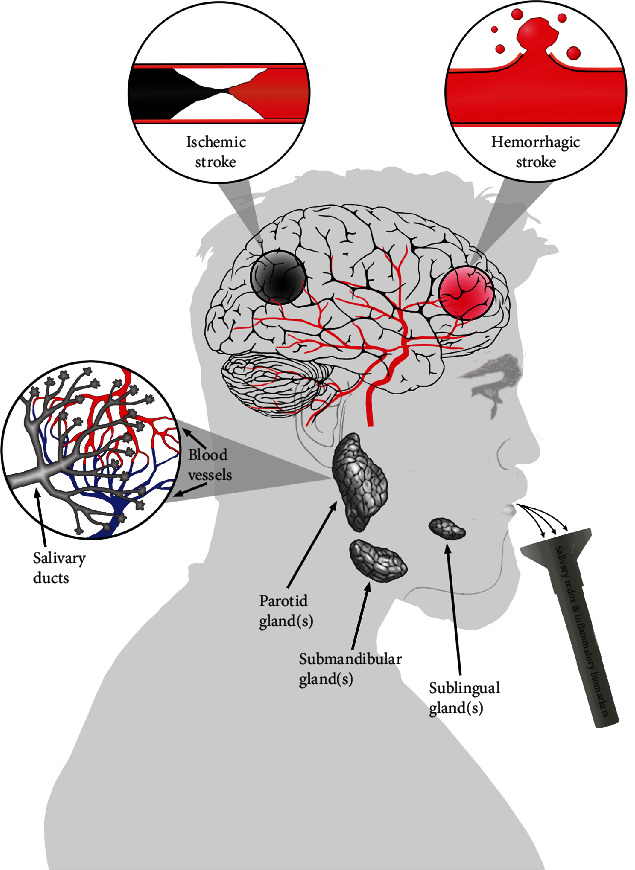
Saliva as a source of redox/inflammatory biomarkers in stroke patients. Recent reports indicate the usefulness of salivary redox biomarkers in many systemic diseases, including ischemic and hemorrhagic stroke. Saliva secreted by the parotid, submandibular, and sublingual glands is a plasma filtrate that is rich in antioxidants and cellular oxidation products. In general, concentrations of many redox biomarkers in saliva reflect their levels in blood.

The use of saliva in laboratory medicine is not limited to the diagnosis of oral diseases. Recent findings indicate the usefulness of salivary redox biomarkers in many systemic diseases, including ischemic and hemorrhagic strokes (Table 2, Figure 3) [22–26, 111]. In general, salivary levels of oxidative stress biomarkers reflect their blood concentrations as evidenced by the high saliva-plasma correlation coefficients. Saliva has notable superiorities over other biofluids. The low cost, high durability, and noninvasive, painless, and hassle-free collection, particularly from the elderly and disabled patients, demonstrate its unquestionable advantages. In stroke cases, salivary redox biomarkers also correlate with the degree of disease progression [22]. Thus, salivary oxidative stress indexes could potentially be used to monitor the severity of the stroke. Importantly, salivary antioxidant concentration/activity in stroke patients does not depend on gender or salivary secretion [22]. Therefore, measurement of such markers may allow for the prediction of stroke outcome and help in elucidating pathophysiological mechanisms mediating injury of the brain and in choosing adequate therapy in a very early stage of the disorder [12]. The diagnostic utility of redox biomarkers in stroke diagnostics/prediction was also confirmed by the ROC analysis [22].

Salivary antioxidants, particularly reduced glutathione, appear to be of high diagnostic usefulness. This biomarker with high specificity and accuracy distinguishes stroke patients from controls but also correlates with cognitive impairment, as well as static and dynamic balance disorders [22, 23]. Importantly, this parameter does not depend on age, sex, or salivary gland function. Thus, salivary GSH may be a potential biomarker of disease severity. On the other hand, salivary products of protein oxidation, glycation, and nitration do not reflect changes at the systemic level. These biomarkers rather provide information about the degree of salivary gland hypofunction, which is found in some stroke patients [22, 24]. Although salivary redox biomarkers assessed to date have high sensitivity and specificity, none of them differentiate ischemic from hemorrhagic stroke. Evaluation of salivary inflammatory biomarkers also does not appear to be clinically relevant [111].

Further clinical trials evaluating salivary redox homeostasis on a larger patient population are needed. Development of noninvasive, rapid, and ambulatory saliva-based screening tests could greatly facilitate early and comprehensive diagnosis of stroke in the future. Optimal laboratory diagnostic tools, including salivary biomarkers, need to be reproducibly measured with standardized and widely available techniques. Moreover, they should be straightforward and have the appropriate specificity and sensitivity. It is also necessary to standardize saliva collection protocols and to estimate reference ranges for salivary redox biomarkers.

Numerous studies have shown that troponins may also be promising biomarkers in stroke diagnostics [218, 219]. They are considered a predictor of stroke severity, prognosis, and mortality, both during hospitalization and in long-term follow-up. An increase in troponins in patients with an acute stroke nearly doubles the risk of mortality at 5 years [218, 219]. Since troponins can be measured in saliva samples, it is advisable to evaluate them in stroke patients [220, 221]. Noninvasive diagnostics using saliva may be a new strategy in subjects with stroke.

In summary, this review concerning salivary redox biomarkers in cerebral stroke patients shows their prospective usefulness in clinical practice. They may be potentially used in diagnosis and as indicators of disease progression.

## 8. Conclusions

1. Salivary redox biomolecules (especially reduced glutathione) may be potential biomarkers in stroke diagnostics
2. There is a need for further studies of salivary redox/inflammatory biomarkers on a larger population of stroke patients, as well as a more in-depth evaluation of saliva-blood relationships

## Figures and Tables

**Table 1:** Inclusion and exclusion criteria.

Inclusion criteria	Exclusion criteria
(1) Articles written in English only (2) Publications on salivary biomarkers of oxidative stress and inflammation in stroke patients (3) Results obtained from experiments participated by humans as well as experimental works, including *in vitro* tests (4) Clinical trials conducted on a group of at least 20 people (5) Meta-analyses	(1) Publications written in a language other than English (2) Clinical studies performed on a group of fewer than 20 people (3) Surveys (4) Case studies

**Table 2:** Salivary redox and inflammatory biomarkers in stroke patients.

Study design				Results			References
Study group	Control group	Smokers/PD	Saliva collection				
				NWS (vs. control group)	SWS (vs. control group)	Comments	
*Redox biomarkers*							
30 patients (18 men and 12 women) with acute hemorrhagic (30%) and ischemic (70%) stroke	30 age- and sex-matched healthy controls	No/no	NWS: expectorating saliva (for 15 min) into a test tube placed in a receptacle containing ice, centrifugation (3000 × g, 4°C, 20 min) SWS: citric acid (2%) stimulation every 30 s for 5 min, expectorating saliva (for 5 min) into a test tube placed in a receptacle containing ice, centrifugation (3000 × g, 4°C, 20 min)	Antioxidants: ↑Px, ↑CAT, ↑SOD, ↑UA (colorimetric methods) Redox status: ↑TOS (colorimetric method) Oxidative damage: ↑AOPP, ↑LOOH (colorimetric methods) Protein glycation: ↑AGE (fluorimetric methods)	Antioxidants: ↑Px, ↑CAT, ↑UA, ↓GSH (colorimetric methods) Redox status: ↑TOS, ↑OSI (TOS/TAC) (colorimetric methods) Oxidative damage: ↑AOPP, ↑LOOH (colorimetric methods) Protein glycation: ↑AGE (fluorimetric methods)	Comparison of hemorrhagic and ischemic stroke: no statistical differences between hemorrhagic and ischemic stroke Correlation analysis: GSH SWS correlated positively with dynamic balance abilities in BBS and cognitive function in the ACE III scale; AGE SWS, AOPP SWS, and LOOH SWS correlated negatively with the salivary flow rate Multifactorial regression analysis: GSH SWS does not depend on gender, age, and salivary flow rate ROC analysis: GSH SWS with sensitivity and specificity equal to 100% differentiates the study group from controls (AUC 1.0); high diagnostic utility is also shown by NWS AOPP (sensitivity 83.33%, specificity 76.67%, AUC 0.8711), NWS LOOH (sensitivity 86.67%, specificity 80%, AUC 0.8567), SWS TOS (sensitivity 86.67%, specificity 83.33%, AUC 0.8822), SWS AOPP (sensitivity 96.67%, specificity 93.33%, AUC 0.9911), and SWS LOOH (sensitivity 93.33%, specificity 90%, AUC 0.9833)	[22]
30 patients (19 men and 11 women) with acute hemorrhagic (20%) and ischemic (80%) stroke divided into two subgroups taking into consideration the rate of salivary secretion: normal salivary secretion (*n* = 16) and hyposalivation (*n* = 14)	30 age- and sex-matched healthy controls with normal salivary secretion (NWS flow > 0.2 mL/min)	No/no	NWS: expectorating saliva (for 15 min) into a test tube placed in a receptacle containing ice, centrifugation (3000 × g, 4°C, 20 min) SWS: citric acid (2%) stimulation every 30 s for 5 min, expectorating saliva (for 5 min) into a test tube placed in a receptacle containing ice, centrifugation (3000 × g, 4°C, 20 min)	Protein glycation: ↑Amadori products, ↑AGE (colorimetric/fluorimetric methods) Protein oxidation: ↑PC, ↓total thiols (colorimetric methods) Protein glycoxidation: ↑dityrosine, ↑kynurenine, ↑N-formylkynurenine, ↓tryptophan (colorimetric/fluorimetric methods) Nitrosative stress: ↓NO, ↑peroxynitrite, ↑nitrotyrosine (colorimetric method/ELISA)	Protein glycation: ↑Amadori products, ↑AGE (colorimetric/fluorimetric methods) Protein oxidation: ↓total thiols (colorimetric methods) Protein glycoxidation: ↑N-formylkynurenine, ↓tryptophan (colorimetric/fluorimetric methods) Nitrosative stress: ↑peroxynitrite (colorimetric method)	General comments: protein glycation (NWS: ↑Amadori products, ↑AGE; SWS: ↑AGE), glycoxidation (NWS: ↑dityrosine), and nitrosative stress (NWS: ↓NO, ↑nitrotyrosine; SWS: ↑peroxynitrite) were significantly increased in stroke patients with hyposalivation when compared to stroke cases with normal salivary secretion Comparison of hemorrhagic and ischemic stroke: no statistical differences between hemorrhagic and ischemic stroke Comparison of NWS and SWS: the content of protein glycation, oxidation and glycoxidation products as well as nitrosative stress biomarkers was significantly higher in NWS compared to SWS in both control and stroke patients Correlation analysis: NWS Amadori products, NWS AGE, NWS PC, NWS dityrosine, NWS kynurenine, NWS N-formylkynurenine, NWS NO, and NWS nitrotyrosine correlated negatively with the salivary flow rate; NWS total thiols correlated positively with the NWS flow. Such changes were not observed in SWS of stroke patients	[24]
Study group: 50 patients (24 men and 26 women) with ischemic stroke Risk groups: 25 patients (12 men and 13 women) with hypertension; 25 patients (11 men and 14 women) with type 2 diabetes	25 age- and sex-matched healthy controls (12 men and 13 women)	ND/no	Expectorating saliva (for 5 min) into a test tube placed in a receptacle containing ice, centrifugation (3000 × g, 4°C, 5 min)	Antioxidants: ↑SOD, ↑UA, ↓GSH (colorimetric methods) Oxidative damage: ↑MDA (colorimetric method)		Serum analysis: ↑SOD, ↑UA, ↓GSH, ↑MDA vs. control group ROC analysis: salivary MDA with a higher accuracy rate (92%) than that of serum values (81%) differentiates healthy individuals from those with ischemic stroke (salivary AUC 0.969, serum AUC 0.885); high diagnostic utility is also shown by salivary (accuracy 89.3%, AUC 0.95) and serum (accuracy 89.3%, AUC 0.927) UA, salivary (accuracy 89.3%, AUC 0.918) and serum (accuracy 80%, AUC 0.838) SOD, and salivary (accuracy 81%, AUC 0.669) and serum (accuracy 80%, AUC 0.912) GSH Prediction analysis: for predicting stroke in diabetic patients, the most valid parameters are salivary UA (AUC 0.709) and salivary SOD (AUC 0.679); the most valid parameters in predicting ischemic stroke from ischemic heart disease are salivary UA (AUC 0.66) and salivary GSH (AUC 0.623); to differentiate stroke from the whole risk group, the most valid parameters are salivary MDA (AUC 0.705) and salivary SOD (AUC 0.661)	[23]
Study group: 50 patients (24 men and 26 women) with ischemic stroke Risk groups: 25 patients (12 men and 13 women) with hypertension; 25 patients (11 men and 14 women) with type 2 diabetes	25 age- and sex-matched healthy controls (12 men and 13 women)	ND/no	Expectorating saliva (for 5 min) into a test tube placed in a receptacle containing ice, centrifugation (3000 × g, 4°C, 5 min)	↑NSE (ELISA)		Serum analysis: ↑NSE vs. control ROC analysis: salivary NSE differentiates stroke patients with controls (AUC 0.763) as well as stroke cases from negative controls (patients with hypertension and type 2 diabetes; AUC 0.825)	[25]

*Inflammatory biomarkers*							
98 patients (53 men and 45 women) with acute ischemic stroke	100 age- and sex-matched healthy controls	Yes/yes	Chewing a piece of paraffin for 2 min	↓MMP-8 ↓MPO ↓IL-1*β*		Serum analysis: ↑MMP-8, ↑MPO vs. control	[111]

Abbreviations: ACE III: Addenbrooke's Cognitive Examination III scale; AGE: advanced glycation end products; AOPP: advanced oxidation protein products; AUC: area under the curve; CAT: catalase; ELISA: enzyme-linked immunosorbent assay; GSH: reduced glutathione; IL-1*β*: interleukin-1*β*; LOOH: lipid hydroperoxides; MDA: malondialdehyde; MMP-8: matrix metalloproteinase-8; MPO: myeloperoxidase; NO: nitric oxide; NSE: neuron-specific enolase; NWS: nonstimulated whole saliva; OSI: oxidative stress index; PC: protein carbonyl groups; PD: periodontal disease; Px: salivary peroxidase; SOD: superoxide dismutase; SWS: stimulated whole saliva; TAC: total antioxidant capacity; TOS: total oxidant status; UA: uric acid.

## Data Availability

The article contains complete data used to support the findings of this study.

## Abbreviations

ACE III: Addenbrooke's Cognitive Examination III
ADP: Adenosine diphosphate (ADP)
ALS: Amyotrophic lateral sclerosis
AOPP: Advanced oxidation protein products
ATP: Adenosine triphosphate
AUC: Area under the curve
BBB: Blood-brain barrier
BBS: Berg balance scale
Br^−^: Bromides
CAT: Catalase
DNA: Deoxyribonucleic acid
GPx: Glutathione peroxidase
GSH: Reduced glutathione
ICH: Intracerebral hemorrhage
iNOS: Inducible nitric oxide synthase
LOOH: Total hydroperoxides
MDA: Malondialdehyde
MPO: Myeloperoxidase
mROS: Mitochondrial reactive oxygen species
Na^+^/K^+^-ATPase: Sodium-potassium pump
NADPH: Nicotinamide adenine dinucleotide phosphate (NADPH)
nNOS: Neuronal NO synthase
NOX: Nicotinamide adenine dinucleotide phosphate (NADPH) oxidase
NSE: Neuron-specific enolase (2-phospho-D-glycerate hydrolase)
NWS: Nonstimulated whole saliva
PC: Protein carbonyls
PEP: Phosphoenolpyruvate
PGA: 2-Phospho-D-glycerate
Px: Salivary peroxidase system
RNS: Reactive nitrogen species
ROS: Reactive oxygen species
SAH: Subarachnoid hemorrhage
SCN^−^: Thiocyanates
SOD: Superoxide dismutase
SWS: Stimulated whole saliva
TAC: Total antioxidant capacity
TOS: Total oxidant capacity
UA: Uric acid
XO: Xanthine oxidase.
